# Supplementary material for: Plasma Metabolomics to Evaluate Progression of Necrotising Enterocolitis in Preterm Pigs
Source: Metabolites. 2021 Apr 29;11(5):283. doi: 10.3390/metabo11050283 (PMC8146597; doi:10.3390/metabo11050283)
Supplement: Supplementary file 1 [file metabolites-11-00283-s001.zip › metabolites-1157224-supplementary.pdf]

Supporting Information for Publication

Plasma Metabolomics to Evaluate Progression of Necrotising Enterocolitis in Preterm Pigs

Yan-Nan Jiang,[1] Yong-Xin Ye,[1] Per Torp Sangild,[2,3,4] Thomas Thymann,[2] Søren Balling Engelsen,[5] Bekzod Khakimov,[5] Ping-Ping Jiang[1,2]

1. School of Public Health, Sun Yat-sen University, Guangzhou, PR China,
2. Section for Comparative Paediatrics and Nutrition, Department of Veterinary and Animal Sciences, University of Copenhagen, Frederiksberg, Denmark,
3. Department of Neonatology, Rigshospitalet, Copenhagen, Denmark,
4. Department of Pediatrics, Odense University Hospital, Odense, Denmark,
5. Department of Food Science, University of Copenhagen, Frederiksberg, Denmark.

Table S1. Body weight and plasma hepatic enzyme levels of pigs included in the NMR analysis,

Table S2. NEC scores and the most affected gut region of pigs,

Table S3. All metabolites annotated with abundance in the antibiotic treatment groups,

Table S4. All metabolites annotated with abundance in NEC severity groups,

Table S5. Antibiotic regimens used,

Table S6. Nutritional composition of the parenteral and enteral nutrition used,

Figure S1. Distribution of NEC severity in the antibiotic treatment groups,

Figure S2. PCA score plots of the metabolomic data.

Table S1. Body weight and plasma hepatic enzyme levels of pigs included in the NMR analysis

	Abundance by antibiotic treatment (mean ± SEM, mM)			*P*-value[1]			Abundance by NEC severity (mean ± SEM, mM)			*P*-value[1]		
	CON	PAR	ORA	PAR vs CON	ORA vs CON	ORA vs PAR	No-NEC	Mild-NEC	Severe-NEC	Mild-NEC vs No-NEC	Severe-NEC vs No-NEC	Severe-NEC vs Mild-NEC
BW[2] at birth (g)	894 ± 56	916 ± 44	916 ± 56	0.53	0.99	0.50	934 ± 36	847 ± 57	894 ± 82	0.13	0.46	0.75
BW on Day 5 (g)	948 ± 67	1031 ± 53	1005 ± 68	0.09	0.91	0.23	1026 ± 43	935 ± 70	965 ± 112	0.07	0.61	0.54
BW gain (Day 0-5, g)	58.8 ± 12.2	98.8 ± 10.0	89.4 ± 15.7	0.02	0.59	0.31	91.2 ± 9.2	88.0 ± 16.1	55.2 ± 18.5	0.59	0.16	0.74
BW gain (g/d)	14.6 ± 3.0	24.7 ± 2.5	22.4 ± 3.9	0.02	0.59	0.31	22.8 ± 2.3	22.0 ± 4.0	13.8 ± 4.6	0.59	0.16	0.74
Adj. BW gain (g/kg/d)	14.4 ± 2.9	23.7 ± 1.7	20.2 ± 3.2	0.03	0.75	0.24	21.1 ± 1.8	22.2 ± 3.7	13.2 ± 3.8	0.87	0.15	0.47
ALP (U/L)	3097 ± 302	2773 ± 173	2590 ± 329	0.75	1	0.79	2524 ± 191	3084 ± 324	3377 ± 360	0.28	0.13	0.95
ALT (U/L)	19.9 ± 1.5	20.3 ± 1.9	17.5 ± 0.6	0.50	0.99	0.64	17.8 ± 0.7	18.4 ± 0.9	23.7 ± 3.0	0.93	0.03	0.03
AST (U/L)	46.1 ± 10.8	93.8 ± 36.2	55.6 ± 30.1	0.09	0.53	0.70	53.1 ± 18.3	41.9 ± 10.4	121.6 ± 54.2	0.78	0.11	0.04
GGT (U/L)	26.9 ± 4.1	25.9 ± 4.0	21.7 ± 2.2	0.71	0.40	0.83	19.8 ± 1.7	27.0 ± 3.9	36.6 ± 6.3	0.58	< 0.01	0.07

[1]*P*-values calculated by Tukey test for pairwise comparison based on linear mixed-effect model; [2]BW, body weight.

Table S2. NEC scores and the most affected GIT region of pigs

Pig	Antibiotic Treatment	NEC score	most affected GIT region	Pig	Antibiotic Treatment	NEC score	most affected GIT region	Pig	Antibiotic Treatment	NEC score	most affected GIT region
P01	CON	6	stomach; colon	P05	PAR	1	-	P06	ORA	1	-
P02	CON	1	-	P09	PAR	4	stomach	P10	ORA	1	-
P04	CON	5	stomach; colon	P12	PAR	1	-	P15	ORA	2	-
P08	CON	5	colon	P14	PAR	4	colon	P16	ORA	2	-
P11	CON	1	-	P21	PAR	3	proximal intestine	P17	ORA	2	-
P13	CON	1	-	P24	PAR	1	-	P18	ORA	1	-
P20	CON	2	-	P27	PAR	3	proximal intestine	P19	ORA	2	-
P23	CON	5	stomach; distal intestine; colon	P28	PAR	5	distal intestine	P22	ORA	1	-
P26	CON	2	-	P29	PAR	3	stomach; proximal intestine; distal intestine	P25	ORA	1	-
P37	CON	6	stomach; distal intestine; colon	P33	PAR	1	-	P30	ORA	2	-
P38	CON	4	colon	P34	PAR	1	-	P31	ORA	1	-
P39	CON	5	colon	P35	PAR	1	-	P32	ORA	1	-
P40	CON	5	colon	P36	PAR	6	mid-intestine; distal intestine; colon	P41	ORA	1	-
P44	CON	3	colon	P42	PAR	5	colon	P47	ORA	1	-
P45	CON	2	-	P43	PAR	4	colon	P49	ORA	1	-
-	-	-	-	P46	PAR	2	-	-	-	-	-
-	-	-	-	P48	PAR	3	colon	-	-	-	-

Table S3. All metabolites annotated with abundance in the antibiotic treatment groups

Metabolite	Molecular formula	Chemical shift (δ, ppm)	Abundance by antibiotics treatment (mean ± SEM, mM)			Effect size		
			CON	PAR	ORA	PAR - CON	ORA - CON	ORA - PAR
Cholesterol	$C_{27}H_{46}O$	0.679	3.67 ± 0.24	3.71 ± 0.34	3.85 ± 0.10	-0.27	-0.28	0.00
Isoleucine	$C_6H_{13}NO_2$	0.960	1.01 ± 0.05	1.02 ± 0.05	1.02 ± 0.04	0.01	0.24	0.23
Leucine	$C_6H_{13}NO_2$	0.983	0.52 ± 0.06	0.55 ± 0.05	0.51 ± 0.04	0.00	0.02	0.03
Valine	$C_5H_{11}NO_2$	1.006	1.44 ± 0.10	1.51 ± 0.08	1.47 ± 0.07	0.04	-0.01	-0.05
Isobutyric acid	$C_4H_7O_2^-$	1.100	0.09 ± 0.01	0.09 ± 0.01	0.10 ± 0.01	-0.31	-0.29	0.02
Ethanol	C_2H_5OH	1.200	0.45 ± 0.05	0.57 ± 0.03	0.46 ± 0.03	0.76	-0.23	-0.99
3-Hydroxybutyric acid	$C_4H_8O_3$	1.229	0.04 ± 0.01	0.05 ± 0.02	0.03 ± 0.01	0.21	-0.68	-0.89
Lactic acid	$C_3H_6O_3$	1.345	3.72 ± 0.90	4.51 ± 1.14	2.91 ± 0.42	0.48	0.07	-0.41
Alanine	$C_3H_7NO_2$	1.508	0.97 ± 0.10	1.18 ± 0.24	0.90 ± 0.08	0.60	0.26	-0.34
Acetic acid	$C_2H_3O_2^-$	1.940	0.16 ± 0.04	0.15 ± 0.02	0.12 ± 0.01	0.00	-0.45	-0.45
Acetoacetic acid	$C_4H_6O_3$	2.295	0.05 ± 0.00	0.05 ± 0.01	0.05 ± 0.00	-0.19	-0.16	0.03
Pyruvate	$C_3H_4O_3$	2.395	0.15 ± 0.02	0.15 ± 0.02	0.13 ± 0.02	0.26	0.09	-0.17
Citrate	$C_6H_8O_7$	2.550	0.41 ± 0.04	0.54 ± 0.06	0.46 ± 0.03	0.71	-0.15	-0.86
Creatine	$C_4H_9N_3O_2$	3.054	0.18 ± 0.02	0.20 ± 0.02	0.27 ± 0.05	0.17	0.31	0.14
Creatinine	$C_4H_7N_3O$	3.063	0.91 ± 0.11	0.99 ± 0.13	0.90 ± 0.08	0.39	0.38	-0.01
Methylsulfonylmethane (MSM/DMSO2)	$C_2H_6O_2S$	3.175	0.00 ± 0.00	0.00 ± 0.00	0.00 ± 0.00	-0.46	-0.01	0.45
Trimethylamine N-oxide (TMAO)	C_3H_9NO	3.257	0.41 ± 0.02	0.42 ± 0.03	0.45 ± 0.02	0.03	0.14	0.11
Myo-inositol	$C_6H_{12}O_6$	3.309	11.50 ± 1.65	9.86 ± 0.83	7.90 ± 1.02	0.17	-0.13	-0.30
Methanol	CH_3OH	3.378	0.13 ± 0.01	0.11 ± 0.01	0.10 ± 0.01	-0.37	-0.06	0.31
Tyrosine	$C_9H_{11}NO_3$	3.933	3.16 ± 0.22	3.66 ± 0.36	3.99 ± 0.19	-0.30	0.02	0.32
3-Phenyllactic acid	$C_9H_{10}O_3$	4.530	0.38 ± 0.04	1.32 ± 0.03	1.70 ± 0.06	1.66	2.33	0.68

Glucose	$C_6H_{12}O_6$	5.257	4.10 ± 0.29	4.48 ± 0.46	4.57 ± 0.31	-0.25	-0.22	0.03
1-Methylhistidine	$C_7H_{11}N_3O_2$	7.015	0.18 ± 0.02	0.18 ± 0.02	0.16 ± 0.02	0.18	-0.39	-0.58
Histidine	$C_6H_9N_3O_2$	7.080	0.16 ± 0.01	0.19 ± 0.02	0.16 ± 0.01	0.69	0.28	-0.41
Tryptophan	$C_{11}H_{12}N_2O_2$	7.212	0.29 ± 0.03	0.41 ± 0.07	0.53 ± 0.09	0.46	0.99	0.54
Phenylalanine	$C_9H_{11}NO_2$	7.350	0.19 ± 0.02	0.24 ± 0.02	0.22 ± 0.01	0.80	0.64	-0.16
Formic acid	$HCOO^-$	8.481	0.26 ± 0.11	0.18 ± 0.04	0.07 ± 0.01	0.44	-0.50	-0.94

Table S4. All metabolites annotated with abundance in NEC severity groups

Metabolite	Molecular formula	Chemical shift (δ, ppm)	Abundance by NEC severity (mean ± SEM, mM)			Effect size		
			No-NEC	Mild-NEC	Severe-NEC	Mild-NEC vs No-NEC	Severe-NEC vs No-NEC	Severe-NEC vs Mild-NEC
Cholesterol	$C_{27}H_{46}O$	0.679	3.91 ± 0.13	4.00 ± 0.36	3.04 ± 0.44	0.09	-1.01	-1.10
Isoleucine	$C_6H_{13}NO_2$	0.960	1.00 ± 0.03	1.05 ± 0.09	1.04 ± 0.07	0.43	0.35	-0.08
Leucine	$C_6H_{13}NO_2$	0.983	0.51 ± 0.03	0.62 ± 0.08	0.50 ± 0.08	0.58	-0.03	-0.61
Valine	$C_5H_{11}NO_2$	1.006	1.47 ± 0.05	1.57 ± 0.14	1.39 ± 0.13	0.28	-0.27	-0.55
Isobutyric acid	$C_4H_7O_2^-$	1.100	0.10 ± 0.00	0.10 ± 0.01	0.08 ± 0.01	-0.17	-1.10	-0.93
Ethanol	C_2H_5OH	1.200	0.50 ± 0.03	0.53 ± 0.04	0.44 ± 0.05	-0.06	-0.33	-0.27
3-Hydroxybutyric acid	$C_4H_8O_3$	1.229	0.05 ± 0.01	0.04 ± 0.01	0.01 ± 0.01	-0.76	-1.11	-0.35
Lactic acid	$C_3H_6O_3$	1.345	3.23 ± 0.41	3.29 ± 0.94	5.61 ± 1.96	-0.20	0.69	0.89
Alanine	$C_3H_7NO_2$	1.508	0.92 ± 0.05	0.93 ± 0.09	1.40 ± 0.42	-0.16	0.85	1.01
Acetic acid	$C_2H_3O_2^-$	1.940	0.13 ± 0.02	0.13 ± 0.01	0.18 ± 0.03	-0.26	0.29	0.56
Acetoacetic acid	$C_4H_6O_3$	2.295	0.05 ± 0.00	0.06 ± 0.01	0.04 ± 0.00	0.53	-0.38	-0.91
Pyruvate	$C_3H_4O_3$	2.395	0.13 ± 0.01	0.13 ± 0.02	0.20 ± 0.04	-0.12	0.86	0.99
Citrate	$C_6H_8O_7$	2.550	0.51 ± 0.04	0.46 ± 0.06	0.39 ± 0.03	-0.75	-0.78	-0.02
Creatine	$C_4H_9N_3O_2$	3.054	0.24 ± 0.03	0.21 ± 0.02	0.15 ± 0.03	0.02	-0.84	-0.85
Creatinine	$C_4H_7N_3O$	3.063	0.87 ± 0.05	0.91 ± 0.14	1.14 ± 0.22	0.09	0.82	0.73
Methylsulfonylmethane (MSM/DMSO2)	$C_2H_6O_2S$	3.175	0.00 ± 0.00	0.00 ± 0.00	0.00 ± 0.00	-0.23	-0.08	0.14
Trimethylamine N-oxide (TMAO)	C_3H_9NO	3.257	0.45 ± 0.02	0.42 ± 0.03	0.39 ± 0.03	-0.24	-0.51	-0.26
Myo-inositol	$C_6H_{12}O_6$	3.309	8.37 ± 0.72	8.93 ± 1.05	14.40 ± 1.93	0.12	1.24	1.11
Methanol	CH_3OH	3.378	0.10 ± 0.01	0.12 ± 0.01	0.14 ± 0.01	0.64	1.02	0.38
Tyrosine	$C_9H_{11}NO_3$	3.933	3.84 ± 0.17	3.84 ± 0.35	2.73 ± 0.45	0.15	-1.07	-1.22
3-Phenyllactic acid	$C_9H_{10}O_3$	4.530	1.31 ± 0.11	1.08 ± 0.12	0.72 ± 0.18	-0.06	0.11	0.16

Glucose	$C_6H_{12}O_6$	5.257	4.64 ± 0.24	4.39 ± 0.52	3.67 ± 0.55	-0.15	-0.97	-0.82
1-Methylhistidine	$C_7H_{11}N_3O_2$	7.015	0.17 ± 0.01	0.16 ± 0.02	0.19 ± 0.02	-0.65	-0.11	0.54
Histidine	$C_6H_9N_3O_2$	7.080	0.16 ± 0.01	0.15 ± 0.01	0.21 ± 0.04	-0.35	0.75	1.10
Tryptophan	$C_{11}H_{12}N_2O_2$	7.212	0.43 ± 0.05	0.40 ± 0.07	0.35 ± 0.11	0.18	0.20	0.03
Phenylalanine	$C_9H_{11}NO_2$	7.350	0.22 ± 0.01	0.24 ± 0.02	0.22 ± 0.03	0.27	0.27	0.00
Formic acid	$HCOO^-$	8.481	0.13 ± 0.04	0.10 ± 0.01	0.35 ± 0.14	-0.37	0.73	1.10

Table S5. Antibiotic regimens used

groups	Antibiotic regimens
PAR	Ampicillin (Pentrexyl; Bristol-Myers Squibb, Solna, Sweden; 30 mg/kg birth weight three times daily), gentamicin (B. Braun Medical, Melsungen, Germany; 2.5 mg/kg BW two times daily), and metronidazole (B. Braun Medical; 10 mg/kg BW three times daily)
ORA	Ampicillin (Penbritin; Chemidex Pharma, Surrey, United Kingdom; 30 mg/kg birth weight three times daily), gentamicin (Gentocin Vet; ScanVet, Fredensborg, Denmark; 2.5 mg/kg BW two times daily), and metronidazole (Flagyl; Sanofi Aventis, Hørsholm, Denmark; 10 mg/kg BW three times daily)

Table S6. Nutritional composition of the parenteral and enteral nutrition used

**Parenteral nutrition**	**Content /L**
Energy	3,123 kJ
Non-protein energy	2,373 kJ
Glucose	72 g
Lipids	31 g
Nitrogen	6.4 g
Amino acids	45 g
Sodium	40 mmol
Potassium	28 mmol
Magnesium	3.2 mmol
Calcium	3.2 mmol
Phosphate	12 mmol
Osmolality	1,540 mosmol

**Enteral nutrition**	**Content /L**
Energy	4,140 kJ
Protein[1]	64 g
Carbohydrate2	45 g
Fat	61 g
Saturated fat	44 g
Monounsaturated fat	10 g
Polyunsaturated fat	4 g
Sodium	0.30 g
Potassium	0.64 g
Calcium	0.59 g
Phosphorus	0.42 g

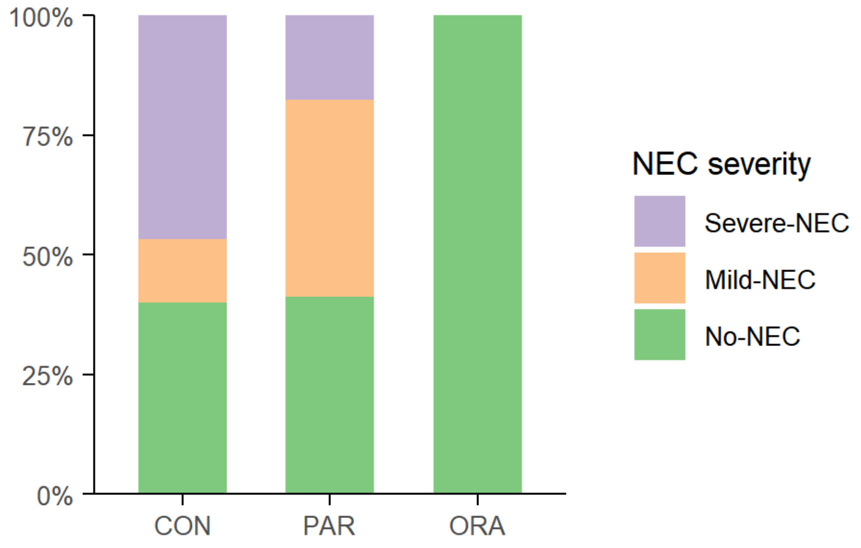

Figure S1. Distribution of NEC severity grouping in the antibiotic treatment groups

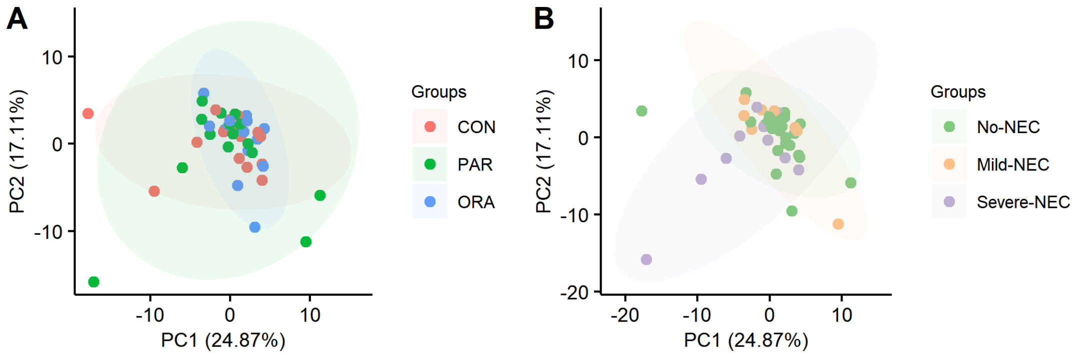

Figure S2. PCA score plots of the metabolomic data
